# Supplementary material for: Eight New Sedum Plastomes: Comprehensive Analyses and Phylogenetic Implications
Source: Genes (Basel). 2025 Jun 28;16(7):761. doi: 10.3390/genes16070761 (PMC12294699; doi:10.3390/genes16070761)
Supplement: Supplementary file 1 [file genes-16-00761-s001.zip › genes-3726588-supplementary.pdf]

Supplementary files

Table S1. Species of plastomes examined in this study.

Table S2. Gene content of the plastomes of eight *Sedum* species.

Table S3. Length, base composition, and skew of the eight *Sedum* plastomes.

Table S4. RSCU values of the eight *Sedum* plastomes.

Table S5. SSRs analysis of the eight *Sedum* plastomes.

Table S6. Predictive RNA editing sites in the eight *Sedum* plastomes.

Table S1

Species of plastomes examined in this study.

Order	Family	Species	Accession No.
Saxifragales	Altingiaceae	*Altingia chinensis*	NC_047288.1
Saxifragales	Altingiaceae	*Altingia excelsa*	NC_048995.1
Saxifragales	Altingiaceae	*Altingia yunnanensis*	NC_048981.1
Saxifragales	Altingiaceae	*Liquidambar acalycina* *	NC_046936.1
Saxifragales	Altingiaceae	*Liquidambar formosana*	NC_023092.1
Saxifragales	Altingiaceae	*Liquidambar orientalis*	NC_046937.1
Saxifragales	Altingiaceae	*Liquidambar styraciflua*	MT079215.1
Saxifragales	Cercidiphyllaceae	*Cercidiphyllum japonicum* *	NC_037940.1
Saxifragales	Cercidiphyllaceae	*Cercidiphyllum magnificum*	NC_046692.1
Saxifragales	Crassulaceae	*Aeonium arboreum voucher SZ* *	MW206792.1
Saxifragales	Crassulaceae	*Bryophyllum daigremontianum*	MT954417.1
Saxifragales	Crassulaceae	*Cotyledon tomentosa* *	MW206793.1
Saxifragales	Crassulaceae	*Crassula perforata*	MW206794 .1
Saxifragales	Crassulaceae	*Graptopetalum amethystinum* *	MW206795.1
Saxifragales	Crassulaceae	*Hylotelephium ewersii* *	MN794014.1
Saxifragales	Crassulaceae	*Hylotelephium verticillatum*	MT558730.1
Saxifragales	Crassulaceae	*Kalanchoe fedtschenkoi*	MW206796.1
Saxifragales	Crassulaceae	*Kalanchoe tomentosa*	MN794319.1
Saxifragales	Crassulaceae	*Orostachys fimbriata*	MW206797.1
Saxifragales	Crassulaceae	*Orostachys japonica* *	MN794320.1
Saxifragales	Crassulaceae	*Pachyphytum compactum*	MW206798.1
Saxifragales	Crassulaceae	*Phedimus aizoon*	MN794321.1

Order	Family	Species	Accession No.
Saxifragales	Crassulaceae	*Phedimus kamtschaticus*	NC_037946.1
Saxifragales	Crassulaceae	*Rhodiola crenulata*	NC_050881.1
Saxifragales	Crassulaceae	*Rhodiola dumulosa* *	MN794323.1
Saxifragales	Crassulaceae	*Rhodiola fastigiata*	MN794324.1
Saxifragales	Crassulaceae	*Rhodiola hobsonii*	MN794325.1
Saxifragales	Crassulaceae	*Rhodiola humilis*	MN794326.1
Saxifragales	Crassulaceae	*Rhodiola integrifolia*	MN794327.1
Saxifragales	Crassulaceae	*Rhodiola kirilowii*	MN109979.1
Saxifragales	Crassulaceae	*Rhodiola ovatisepala*	MN794328.1
Saxifragales	Crassulaceae	*Rhodiola prainii*	MN794329.1
Saxifragales	Crassulaceae	*Rhodiola rhodantha*	MN794330.1
Saxifragales	Crassulaceae	*Rhodiola rosea*	NC_041671.1
Saxifragales	Crassulaceae	*Rhodiola sacra*	MN109978.1
Saxifragales	Crassulaceae	*Rhodiola sexifolia*	MN109980.1
Saxifragales	Crassulaceae	*Rhodiola smithii*	MN794331.1
Saxifragales	Crassulaceae	*Rhodiola yunnanensis*	MN794332.1
Saxifragales	Crassulaceae	*Rhodoleia championii*	MK834325.1
Saxifragales	Crassulaceae	*Rosularia alpestris*	MN794333.1
Saxifragales	Crassulaceae	*Sedum emarginatum*	MT680404.1
Saxifragales	Crassulaceae	*Sedum alfredii*	This study
Saxifragales	Crassulaceae	*Sedum burrito*	This study
Saxifragales	Crassulaceae	*Sedum clavatum*	This study
Saxifragales	Crassulaceae	*Sedum dasyphyllum*	This study
Saxifragales	Crassulaceae	*Sedum furfuraceum*	This study
Saxifragales	Crassulaceae	*Sedum hernandezii*	This study

Order	Family	Species	Accession No.
Saxifragales	Crassulaceae	*Sedum makinoi*	This study
Saxifragales	Crassulaceae	*Sedum palmeri*	This study
Saxifragales	Crassulaceae	*Sedum lineare*	MT755626.1
Saxifragales	Crassulaceae	*Sedum oryzifolium*	NC_027837.1
Saxifragales	Crassulaceae	*Sedum plumbizincicola*	MN185459.1
Saxifragales	Crassulaceae	*Sedum sarmentosum*	NC_023085.1
Saxifragales	Crassulaceae	*Sedum takesimense*	NC_026065.1
Saxifragales	Crassulaceae	*Sempervivum tectorum*	MW206799.1
Saxifragales	Crassulaceae	*Sinocrassula densirosulata*	MW206800.1
Saxifragales	Crassulaceae	*Sinocrassula indica*	MN794334.1
Saxifragales	Crassulaceae	*Umbilicus rupestris*	MN794335.1
Saxifragales	Daphniphyllaceae	*Daphniphyllum macropodum*	MN496060.1
Saxifragales	Daphniphyllaceae	*Daphniphyllum oldhamii* *	NC_037883.1
Saxifragales	Grossulariaceae	*Ribes fasciculatum var. chinense*	MH191388.1
Saxifragales	Grossulariaceae	*Ribes nevadense* *	MN496075.1
Saxifragales	Grossulariaceae	*Ribes roezlii*	MN496076.1
Saxifragales	Haloragaceae	*Myriophyllum aquaticum* *	NC_048889.1
Saxifragales	Haloragaceae	*Myriophyllum spicatum*	NC_037885.1
Saxifragales	Hamamelidaceae	*Chunia bucklandioides* *	NC_041163.1
Saxifragales	Hamamelidaceae	*Corylopsis coreana*	NC_040141.1
Saxifragales	Hamamelidaceae	*Corylopsis multiflora var. nivea*	MW043717.1
Saxifragales	Hamamelidaceae	*Corylopsis spicata*	MK942341.1
Saxifragales	Hamamelidaceae	*Disanthus cercidifolius subsp. longipes*	NC_050371.1
Saxifragales	Hamamelidaceae	*Distylium buxifolium*	MW248115.1
Saxifragales	Hamamelidaceae	*Distylium chinense*	MW248112.1

Order	Family	Species	Accession No.
Saxifragales	Hamamelidaceae	*Distylium cuspidatum*	MW248117.1
Saxifragales	Hamamelidaceae	*Distylium dunnianum*	MW248109.1
Saxifragales	Hamamelidaceae	*Distylium elaeagnoides*	MW248120.1
Saxifragales	Hamamelidaceae	*Distylium gracile*	MW248116.1
Saxifragales	Hamamelidaceae	*Distylium lepidotum*	MW248118.1
Saxifragales	Hamamelidaceae	*Distylium macrophyllum*	MN729500.1
Saxifragales	Hamamelidaceae	*Distylium myricoides*	MW248110.1
Saxifragales	Hamamelidaceae	*Distylium pingpienense*	MW248114.1
Saxifragales	Hamamelidaceae	*Distylium racemosum*	MW248113.1
Saxifragales	Hamamelidaceae	*Distylium tsiangii*	MN711651.1
Saxifragales	Hamamelidaceae	*Fortunearia sinensis*	MN496061.1
Saxifragales	Hamamelidaceae	*Hamamelis mollis*	NC_037881.1
Saxifragales	Hamamelidaceae	*Loropetalum chinense var. rubrum*	MW346664.1
Saxifragales	Hamamelidaceae	*Loropetalum subcordatum*	NC_037694.1
Saxifragales	Hamamelidaceae	*Mytilaria laosensis*	NC_048997.1
Saxifragales	Hamamelidaceae	*Parrotia subaequalis*	MG334121.1
Saxifragales	Hamamelidaceae	*Semiliquidambar cathayensis*	MN837678.1
Saxifragales	Hamamelidaceae	*Sinowilsonia henryi*	MF687003.1
Saxifragales	Hamamelidaceae	*Sycopsis sinensis*	MT323104.1
Saxifragales	Iteaceae	*Itea chinensis* *	NC_037884.1
Saxifragales	Paeoniaceae	*Paeonia anomala*	MT210549.1
Saxifragales	Paeoniaceae	*Paeonia brownii*	MH191385.1
Saxifragales	Paeoniaceae	*Paeonia decomposita*	MG571273.1
Saxifragales	Paeoniaceae	*Paeonia delavayi*	MN463100.1
Saxifragales	Paeoniaceae	*Paeonia emodi*	MT210548.1

**Order**	**Family**	**Species**	**Accession No.**
Saxifragales	Paeoniaceae	*Paeonia intermedia*	MT210547.1
Saxifragales	Paeoniaceae	*Paeonia jishanensis*	MT210545.1
Saxifragales	Paeoniaceae	*Paeonia lactiflora* *	NC_040983.1
Saxifragales	Paeoniaceae	*Paeonia ludlowii*	KY817592.1
Saxifragales	Paeoniaceae	*Paeonia lutea*	NC_050331.1
Saxifragales	Paeoniaceae	*Paeonia obovata*	KJ206533.1
Saxifragales	Paeoniaceae	*Paeonia ostii*	MK701990.1
Saxifragales	Paeoniaceae	*Paeonia potaninii*	MK701991.1
Saxifragales	Paeoniaceae	*Paeonia qiui*	MT210544.1
Saxifragales	Paeoniaceae	*Paeonia rockii*	MF488719.1
Saxifragales	Paeoniaceae	*Paeonia suffruticosa*	MH191384.1
Saxifragales	Paeoniaceae	*Paeonia veitchii*	KT894821.1
Saxifragales	Penthoraceae	*Penthorum chinense*	JX436155.1
Saxifragales	Saxifragaceae	*Astilboides tabularis*	NC_051893.1
Saxifragales	Saxifragaceae	*Bergenia scopulosa*	NC_036061.1
Saxifragales	Saxifragaceae	*Boykinia aconitifolia*	MN496058.1
Saxifragales	Saxifragaceae	*Chrysosplenium alternifolium*	NC_051986.1
Saxifragales	Saxifragaceae	*Chrysosplenium aureobracteatum*	NC_039740.1
Saxifragales	Saxifragaceae	*Chrysosplenium flagelliferum*	MN729584.1
Saxifragales	Saxifragaceae	*Chrysosplenium kamtschaticum*	NC_051988.1
Saxifragales	Saxifragaceae	*Chrysosplenium lanuginosum*	MK814607.1
Saxifragales	Saxifragaceae	*Chrysosplenium macrophyllum*	MK973001.1
Saxifragales	Saxifragaceae	*Chrysosplenium ramosum*	MK973002.1
Saxifragales	Saxifragaceae	*Chrysosplenium sinicum*	NC_051987.1
Saxifragales	Saxifragaceae	*Heuchera abramsii*	MN496062.1

Order	Family	Species	Accession No.
Saxifragales	Saxifragaceae	*Heuchera alba*	MN496063.1
Saxifragales	Saxifragaceae	*Heuchera caespitosa*	MN496064.1
Saxifragales	Saxifragaceae	*Heuchera eastwoodiae*	MN496065.1
Saxifragales	Saxifragaceae	*Heuchera grossulariifolia var. grossulariifolia*	MN496066.1
Saxifragales	Saxifragaceae	*Heuchera longipetala var. longipetala*	MN496067.1
Saxifragales	Saxifragaceae	*Heuchera mexicana*	MN496068.1
Saxifragales	Saxifragaceae	*Heuchera parviflora var. saurensis*	KR478645.1
Saxifragales	Saxifragaceae	*Heuchera parvifolia var. utahensis*	MN496069.1
Saxifragales	Saxifragaceae	*Heuchera richardsonii*	NC_042923.1
Saxifragales	Saxifragaceae	*Heuchera villosa*	NC_042924.1
Saxifragales	Saxifragaceae	*Leptarrhena pyrolifolia*	MN496070.1
Saxifragales	Saxifragaceae	*Micranthes melanocentra*	MT740256.1
Saxifragales	Saxifragaceae	*Mitella diphylla*	NC_042925.1
Saxifragales	Saxifragaceae	*Mitella formosana*	NC_042926.1
Saxifragales	Saxifragaceae	*Mukdenia rossii*	NC_037495.1
Saxifragales	Saxifragaceae	*Oresitrophe rupifraga*	NC_037514.1
Saxifragales	Saxifragaceae	*Pectiantia pentandra*	MN496072.1
Saxifragales	Saxifragaceae	*Rodgersia aesculifolia*	MW327540.1
Saxifragales	Saxifragaceae	*Rodgersia sambucifolia*	MN496077.1
Saxifragales	Saxifragaceae	*Saniculiphyllum guangxiense*	MN496078.1
Saxifragales	Saxifragaceae	*Saxifraga sinomontana*	MN104589.1
Saxifragales	Saxifragaceae	*Saxifraga stolonifera*	MH191389.1
Saxifragales	Saxifragaceae	*Tanakaea radicans*	MW300581.1
Saxifragales	Saxifragaceae	*Tiarella cordifolia*	MH708567.1
Saxifragales	Saxifragaceae	*Tiarella polyphylla*	MH708568.1

Order	Family	Species	Accession No.
Saxifragales	Saxifragaceae	*Tiarella trifoliata*	MH708572.1
Vitales	Vitaceae	*Vitis heyneana*	MN389557.1
Vitales	Vitaceae	*Vitis vinifera*	NC_007957.1

* denotes the involved plastomes in IR-junction comparative analysis.

Table S2

Gene content of the plastomes of eight *Sedum* species.

**Gene Category**	**Gene Name**
Subunits of photosystem I	_psaA, psaB, psaC, psaI, psaJ_
Subunits of photosystem II	_psbA, psbB, psbC, psbD, psbE, psbF, psbH, psbI, psbJ, psbK, psbL, psbM, psbN, psbT, psbZ_
Small subunit of ribosome	_rps2, rps3, rps4, rps7(2), rps8, rps11, rps12[*](2), rps14, rps15, rps16[*], rps18, rps19[Ψ]_
Large subunit of ribosome	_rpl2[*](2), rpl14, rpl16[*], rpl20, rpl22, rpl23(2), rpl32, rpl33, rpl36_
Subunits of cytochrome b/fcomplex	_petA, petB[*], petD[*], petG, petL, petN_
Subunits of ATP synthase	_atpA, atpB, atpE, atpF[*], atpH, atpI_
DNA dependent RNA polymerase	_rpoA, rpoB, rpoC1[*], rpoC2_
NADPH dehydrogenase	_ndhA[*],ndhB[*](2), ndhC, ndhD, ndhE, ndhF, ndhG, ndhH, ndhI, ndhJ, ndhK_
Protease	_clpP[*]_
Maturase	_matK_
Envelope membrane protein	_cemA_
Translaton initiation factor	_infA_
Cytochrome c biogenesis	_ccsA_
Subunit Acetyl CoA Carboxylate	_accD_
Subunit of rubisco	_rbcL_
Ribosomal RNAs	_rrn4.5(2), rrn5(2), rrn16(2), rrn23(2)_
Conserved open reading frames	_ycf1[Ψ], ycf2(2), ycf3[*], ycf4_
Transfer RNA	_trnA-UGC[*](2), trnC-GCA, trnD-GUC, trnE-UUC, trnF-GAA, trnfM-CAU, trnG-GCC, trnH-GUG[*], trnI-CAU(2), trnI-GAU[*](2), trnK-UUU[*], trnL-CAA(2), trnL-UAA[*], trnL-UAG, trnM-CAU, trnN-GUU(2), trnP-UGG, trnQ-UUG, trnR-ACC(2), trnR-UCU, trnS-GCU, trnS-GGA, trnS-UGA, trnT-GGU, trnT-UGU, trnV-GAC(2), trnV-UAC[*], trnW-CCA, trnY-GUA_

＊ denotes the introns-contained genes, Ψ denotes the pseudo genes, (2) denotes genes that have two copies.

Table S3

Length, base composition, and skew of the eight *Sedum* plastomes.

a Base composition in the *Sedum alfredii* chloroplast genome.

	T %	C %	A%	G%	Length (bp)
**Total**	31.4	19.2	30.9	18.5	149,486
**LSC**	32.7	18.4	31.6	17.3	81,759
**SSC**	33.9	16.3	34.5	15.3	16,663
**IR**	28.3	22.3	28.7	20.7	25,532
**Protein Coding genes**	31.7	17.5	30.7	20.1	77,514
**1st Position**	24	18.9	30.5	26.8	25,838
**2nd position**	33	20.2	29.2	17.7	25,838
**3rd position**	38	13.5	32.4	15.9	25,838

b Base composition in the *Sedum burrito* chloroplast genome.

	T %	C %	A%	G%	Length (bp)
**Total**	31.3	19.3	30.8	18.6	150,275
**LSC**	32.6	18.5	31.6	17.4	82,013
**SSC**	33.6	16.4	34.4	15.6	16,714
**IR**	28.7	22.3	28.3	20.7	25,774
**Protein Coding genes**	31.6	17.6	30.7	20.2	77,642
**1st Position**	24	18.9	30.4	26.9	25,881
**2nd position**	33	20.2	29.3	17.7	25,881
**3rd position**	38	13.7	32.2	15.9	25,881

c Base composition in the *Sedum clavatum* chloroplast genome.

	T %	C %	A%	G%	Length (bp)
**Total**	31.3	19.3	30.8	18.6	148,618
**LSC**	32.5	18.5	31.5	17.4	80,301
**SSC**	33.7	16.4	34.4	15.5	16,751
**IR**	28.7	22.3	28.3	20.7	25,783
**Protein Coding genes**	31.7	17.5	30.7	20.1	75,669
**1st Position**	24	18.8	30.5	26.9	25,223
**2nd position**	33	20.2	29.2	17.7	25,223
**3rd position**	38	13.6	32.3	15.9	25,223

d Base composition in the *Sedum dasyphyllum* chloroplast genome.

	T %	C %	A%	G%	Length (bp)
**Total**	31.3	19.2	30.9	18.6	150,714
**LSC**	32.6	18.4	31.7	17.4	82,779
**SSC**	33.8	16.4	34.2	15.5	16,657
**IR**	28.7	22.3	28.2	20.8	25,639
**Protein Coding genes**	31.6	17.7	30.4	20.3	75,648
**1st Position**	24	19.0	30.1	27.1	25,216
**2nd position**	33	20.4	29.0	17.8	25,216
**3rd position**	38	13.7	32.0	16.0	25,216

e Base composition in the *Sedum furfuraceum* chloroplast genome.

	T %	C %	A%	G%	Length (bp)
**Total**	31.3	19.3	30.8	18.6	149,554
**LSC**	32.6	18.5	31.5	17.4	81,201
**SSC**	33.7	16.4	34.4	15.6	16,745
**IR**	28.7	22.3	28.3	20.7	25,804
**Protein Coding genes**	31.7	17.6	30.6	20.1	76,365
**1st Position**	24	18.9	30.4	26.8	25,455
**2nd position**	33	20.2	29.3	17.6	25,455
**3rd position**	38	13.6	32.2	15.9	25,455

f Base composition in the *Sedum hernandezii* chloroplast genome.

	T %	C %	A%	G%	Length (bp)
**Total**	31.3	19.3	30.8	18.6	150,153
**LSC**	32.6	18.5	31.6	17.3	81,827
**SSC**	33.7	16.4	34.4	15.5	16,746
**IR**	28.7	22.3	28.3	20.7	25,790
**Protein Coding genes**	31.6	17.6	30.6	20.2	77,637
**1st Position**	24	18.9	30.4	26.9	25,879
**2nd position**	33	20.2	29.3	17.7	25,879
**3rd position**	38	13.6	32.2	15.8	25,879

g Base composition in the *Sedum makinoi* chloroplast genome.

	T %	C %	A%	G%	Length (bp)
**Total**	31.3	19.2	30.9	18.6	148,925
**LSC**	32.7	18.4	31.6	17.3	81,173
**SSC**	33.5	16.4	34.7	15.5	16,680
**IR**	28.8	22.3	28.3	20.7	25,536
**Protein Coding genes**	31.7	17.6	30.7	20.1	76,647
**1st Position**	24	19.0	30.4	26.8	25,549
**2nd position**	33	20.2	29.2	17.6	25,549
**3rd position**	38	13.5	32.4	15.8	25,549

h Base composition in the *Sedum palmeri* chloroplast genome.

	T %	C %	A%	G%	Length (bp)
**Total**	31.3	19.3	30.8	18.6	149,833
**LSC**	32.6	18.5	31.5	17.4	81,615
**SSC**	33.8	16.4	34.4	15.5	16,678
**IR**	28.7	22.3	28.3	20.7	25,770
**Protein Coding genes**	31.7	17.5	30.7	20.1	77,063
**1st Position**	24	18.8	30.5	26.6	25,688
**2nd position**	33	20.1	29.3	17.7	25,688
**3rd position**	38	13.6	32.2	16.0	25,688

Table S4

RSCU values of the eight *Sedum* plastomes.

Codon	Number	RSCU	Codon	Number	RSCU
UUU(F)	8349	1.28	UCU(S)	4243	1.47
UUC(F)	4707	0.72	UCC(S)	2650	0.92
UUA(L)	5752	1.64	UCA(S)	3700	1.28
UUG(L)	4372	1.24	UCG(S)	2075	0.72
CUU(L)	4290	1.22	CCU(P)	2430	1.28
CUC(L)	1795	0.51	CCC(P)	1605	0.84
CUA(L)	2995	0.85	CCA(P)	2173	1.14
CUG(L)	1870	0.53	CCG(P)	1392	0.73
AUU(I)	7618	1.38	ACU(T)	3354	1.37
AUC(I)	3966	0.72	ACC(T)	1898	0.78
AUA(I)	4966	0.9	ACA(T)	3081	1.26
AUG(M)	4337	1	ACG(T)	1462	0.6
GUU(V)	3794	1.48	GCU(A)	3093	1.59
GUC(V)	1552	0.61	GCC(A)	1284	0.66
GUA(V)	3242	1.27	GCA(A)	2373	1.22
GUG(V)	1652	0.65	GCG(A)	1033	0.53
UAU(Y)	6165	1.44	UGU(C)	2442	1.18
UAC(Y)	2423	0.56	UGC(C)	1700	0.82
UAA(*)	2635	1.08	UGA(*)	2589	1.06
UAG(*)	2080	0.85	UGG(W)	3934	1
CAU(H)	3222	1.4	CGU(R)	1914	0.89
CAC(H)	1378	0.6	CGC(R)	837	0.39

Codon	Number	RSCU	Codon	Number	RSCU
CAA(Q)	4768	1.44	CGA(R)	2596	1.21
CAG(Q)	1861	0.56	CGG(R)	1317	0.62
AAU(N)	6896	1.4	AGU(S)	2956	1.02
AAC(N)	2940	0.6	AGC(S)	1685	0.58
AAA(K)	8058	1.41	AGA(R)	4075	1.9
AAG(K)	3371	0.59	AGG(R)	2099	0.98
GAU(D)	5113	1.51	GGU(G)	3329	1.12
GAC(D)	1669	0.49	GGC(G)	1434	0.48
GAA(E)	6335	1.43	GGA(G)	4434	1.49
GAG(E)	2538	0.57	GGG(G)	2671	0.9

a Relative synonymous codon usage (RSCU) in the chloroplast genome of *Sedum alfredii*.

Codon	Number	RSCU	Codon	Number	RSCU
UUU(F)	1000	1.36	UCU(S)	556	1.66
UUC(F)	476	0.64	UCC(S)	305	0.91
UUA(L)	909	1.94	UCA(S)	406	1.21
UUG(L)	534	1.14	UCG(S)	206	0.61
CUU(L)	594	1.27	CCU(P)	395	1.49
CUC(L)	167	0.36	CCC(P)	219	0.83
CUA(L)	402	0.86	CCA(P)	282	1.06
CUG(L)	201	0.43	CCG(P)	165	0.62
AUU(I)	1078	1.48	ACU(T)	563	1.7
AUC(I)	423	0.58	ACC(T)	219	0.66
AUA(I)	688	0.94	ACA(T)	403	1.21
AUG(M)	607	1	ACG(T)	143	0.43
GUU(V)	545	1.52	GCU(A)	603	1.78
GUC(V)	173	0.48	GCC(A)	198	0.59
GUA(V)	519	1.45	GCA(A)	414	1.22
GUG(V)	193	0.54	GCG(A)	138	0.41
UAU(Y)	758	1.64	UGU(C)	217	1.48
UAC(Y)	168	0.36	UGC(C)	76	0.52
UAA(*)	49	1.75	UGA(*)	16	0.57
UAG(*)	19	0.68	UGG(W)	454	1
CAU(H)	477	1.51	CGU(R)	338	1.34
CAC(H)	153	0.49	CGC(R)	112	0.44
CAA(Q)	706	1.54	CGA(R)	366	1.45

Codon	Number	RSCU	Codon	Number	RSCU
CAG(Q)	208	0.46	CGG(R)	107	0.42
AAU(N)	949	1.53	AGU(S)	424	1.26
AAC(N)	288	0.47	AGC(S)	118	0.35
AAA(K)	1059	1.53	AGA(R)	447	1.77
AAG(K)	321	0.47	AGG(R)	149	0.59
GAU(D)	841	1.59	GGU(G)	566	1.29
GAC(D)	216	0.41	GGC(G)	165	0.38
GAA(E)	994	1.5	GGA(G)	702	1.6
GAG(E)	334	0.5	GGG(G)	317	0.72

b Relative synonymous codon usage (RSCU) in the chloroplast genome of *Sedum burrito*.

Codon	Number	RSCU	Codon	Number	RSCU
UUU(F)	920	1.08	UCU(S)	358	1.38
UUC(F)	782	0.92	UCC(S)	274	1.06
UUA(L)	628	1.05	UCA(S)	370	1.43
UUG(L)	862	1.44	UCG(S)	272	1.05
CUU(L)	580	0.97	CCU(P)	197	0.82
CUC(L)	454	0.76	CCC(P)	202	0.84
CUA(L)	585	0.98	CCA(P)	284	1.18
CUG(L)	482	0.81	CCG(P)	276	1.15
AUU(I)	793	1.05	ACU(T)	179	0.85
AUC(I)	667	0.88	ACC(T)	167	0.8
AUA(I)	813	1.07	ACA(T)	309	1.47
AUG(M)	738	1	ACG(T)	184	0.88
GUU(V)	438	1.16	GCU(A)	86	0.72
GUC(V)	280	0.74	GCC(A)	113	0.95
GUA(V)	448	1.19	GCA(A)	145	1.21
GUG(V)	346	0.92	GCG(A)	134	1.12
UAU(Y)	617	1.15	UGU(C)	285	1.16
UAC(Y)	452	0.85	UGC(C)	207	0.84
UAA(*)	656	1.01	UGA(*)	538	0.83
UAG(*)	760	1.17	UGG(W)	489	1
CAU(H)	298	1.12	CGU(R)	87	0.41
CAC(H)	236	0.88	CGC(R)	91	0.43
CAA(Q)	551	1.14	CGA(R)	226	1.06

Codon	Number	RSCU	Codon	Number	RSCU
CAG(Q)	413	0.86	CGG(R)	238	1.12
AAU(N)	641	1.18	AGU(S)	159	0.61
AAC(N)	447	0.82	AGC(S)	120	0.46
AAA(K)	1013	1.17	AGA(R)	352	1.65
AAG(K)	719	0.83	AGG(R)	283	1.33
GAU(D)	363	1.17	GGU(G)	238	0.91
GAC(D)	256	0.83	GGC(G)	157	0.6
GAA(E)	574	1.18	GGA(G)	329	1.26
GAG(E)	396	0.82	GGG(G)	323	1.23

c Relative synonymous codon usage (RSCU) in the chloroplast genome of *Sedum clavatum*.

**Codon**	**Number**	**RSCU**	**Codon**	**Number**	**RSCU**
UUU(F)	920	1.08	UCU(S)	358	1.38
UUC(F)	782	0.92	UCC(S)	274	1.06
UUA(L)	628	1.05	UCA(S)	370	1.43
UUG(L)	862	1.44	UCG(S)	272	1.05
CUU(L)	580	0.97	CCU(P)	197	0.82
CUC(L)	454	0.76	CCC(P)	202	0.84
CUA(L)	585	0.98	CCA(P)	284	1.18
CUG(L)	482	0.81	CCG(P)	276	1.15
AUU(I)	793	1.05	ACU(T)	179	0.85
AUC(I)	667	0.88	ACC(T)	167	0.8
AUA(I)	813	1.07	ACA(T)	309	1.47
AUG(M)	738	1	ACG(T)	184	0.88
GUU(V)	438	1.16	GCU(A)	86	0.72
GUC(V)	280	0.74	GCC(A)	113	0.95
GUA(V)	448	1.19	GCA(A)	145	1.21
GUG(V)	346	0.92	GCG(A)	134	1.12
UAU(Y)	617	1.15	UGU(C)	285	1.16
UAC(Y)	452	0.85	UGC(C)	207	0.84
UAA(*)	656	1.01	UGA(*)	538	0.83
UAG(*)	760	1.17	UGG(W)	489	1
CAU(H)	298	1.12	CGU(R)	87	0.41
CAC(H)	236	0.88	CGC(R)	91	0.43
CAA(Q)	551	1.14	CGA(R)	226	1.06

Codon	Number	RSCU	Codon	Number	RSCU
CAG(Q)	413	0.86	CGG(R)	238	1.12
AAU(N)	641	1.18	AGU(S)	159	0.61
AAC(N)	447	0.82	AGC(S)	120	0.46
AAA(K)	1013	1.17	AGA(R)	352	1.65
AAG(K)	719	0.83	AGG(R)	283	1.33
GAU(D)	363	1.17	GGU(G)	238	0.91
GAC(D)	256	0.83	GGC(G)	157	0.6
GAA(E)	574	1.18	GGA(G)	329	1.26
GAG(E)	396	0.82	GGG(G)	323	1.23

d Relative synonymous codon usage (RSCU) in the chloroplast genome of *Sedum dasyphyllum*.

Codon	Number	RSCU	Codon	Number	RSCU
UUU(F)	977	1.34	UCU(S)	542	1.66
UUC(F)	477	0.66	UCC(S)	297	0.91
UUA(L)	860	1.91	UCA(S)	392	1.2
UUG(L)	527	1.17	UCG(S)	200	0.61
CUU(L)	572	1.27	CCU(P)	387	1.47
CUC(L)	184	0.41	CCC(P)	235	0.89
CUA(L)	378	0.84	CCA(P)	271	1.03
CUG(L)	185	0.41	CCG(P)	160	0.61
AUU(I)	1033	1.47	ACU(T)	537	1.67
AUC(I)	405	0.58	ACC(T)	233	0.72
AUA(I)	668	0.95	ACA(T)	382	1.19
AUG(M)	609	1	ACG(T)	136	0.42
GUU(V)	533	1.51	GCU(A)	611	1.8
GUC(V)	174	0.49	GCC(A)	206	0.61
GUA(V)	509	1.44	GCA(A)	402	1.18
GUG(V)	193	0.55	GCG(A)	141	0.41
UAU(Y)	731	1.64	UGU(C)	214	1.46
UAC(Y)	163	0.36	UGC(C)	79	0.54
UAA(*)	49	1.75	UGA(*)	15	0.54
UAG(*)	20	0.71	UGG(W)	447	1
CAU(H)	469	1.5	CGU(R)	331	1.34
CAC(H)	157	0.5	CGC(R)	107	0.43
CAA(Q)	677	1.54	CGA(R)	373	1.51

Codon	Number	RSCU	Codon	Number	RSCU
CAG(Q)	202	0.46	CGG(R)	105	0.42
AAU(N)	908	1.54	AGU(S)	402	1.23
AAC(N)	274	0.46	AGC(S)	121	0.37
AAA(K)	997	1.51	AGA(R)	420	1.7
AAG(K)	325	0.49	AGG(R)	150	0.61
GAU(D)	810	1.59	GGU(G)	579	1.34
GAC(D)	206	0.41	GGC(G)	146	0.34
GAA(E)	985	1.49	GGA(G)	696	1.61
GAG(E)	335	0.51	GGG(G)	307	0.71

e Relative synonymous codon usage (RSCU) in the chloroplast genome of *Sedum furfuraceum*.

Codon	Number	RSCU	Codon	Number	RSCU
UUU(F)	984	1.33	UCU(S)	546	1.66
UUC(F)	496	0.67	UCC(S)	297	0.9
UUA(L)	881	1.92	UCA(S)	400	1.21
UUG(L)	527	1.15	UCG(S)	205	0.62
CUU(L)	582	1.27	CCU(P)	390	1.49
CUC(L)	169	0.37	CCC(P)	217	0.83
CUA(L)	397	0.87	CCA(P)	274	1.05
CUG(L)	193	0.42	CCG(P)	163	0.62
AUU(I)	1054	1.47	ACU(T)	556	1.71
AUC(I)	423	0.59	ACC(T)	214	0.66
AUA(I)	673	0.94	ACA(T)	393	1.21
AUG(M)	600	1	ACG(T)	134	0.41
GUU(V)	533	1.52	GCU(A)	602	1.79
GUC(V)	167	0.48	GCC(A)	202	0.6
GUA(V)	515	1.47	GCA(A)	410	1.22
GUG(V)	188	0.54	GCG(A)	135	0.4
UAU(Y)	747	1.63	UGU(C)	209	1.46
UAC(Y)	168	0.37	UGC(C)	78	0.54
UAA(*)	46	1.62	UGA(*)	19	0.67
UAG(*)	20	0.71	UGG(W)	456	1
CAU(H)	464	1.49	CGU(R)	332	1.35
CAC(H)	160	0.51	CGC(R)	101	0.41
CAA(Q)	696	1.54	CGA(R)	354	1.44

Codon	Number	RSCU	Codon	Number	RSCU
CAG(Q)	207	0.46	CGG(R)	102	0.41
AAU(N)	945	1.54	AGU(S)	414	1.26
AAC(N)	281	0.46	AGC(S)	114	0.35
AAA(K)	1033	1.52	AGA(R)	443	1.8
AAG(K)	327	0.48	AGG(R)	146	0.59
GAU(D)	825	1.59	GGU(G)	553	1.29
GAC(D)	215	0.41	GGC(G)	167	0.39
GAA(E)	978	1.49	GGA(G)	690	1.61
GAG(E)	337	0.51	GGG(G)	308	0.72

f Relative synonymous codon usage (RSCU) in the chloroplast genome of *Sedum hernandezii*.

Codon	Number	RSCU	Codon	Number	RSCU
UUU(F)	997	1.34	UCU(S)	552	1.65
UUC(F)	493	0.66	UCC(S)	306	0.92
UUA(L)	892	1.92	UCA(S)	405	1.21
UUG(L)	540	1.16	UCG(S)	203	0.61
CUU(L)	589	1.27	CCU(P)	399	1.5
CUC(L)	172	0.37	CCC(P)	222	0.83
CUA(L)	399	0.86	CCA(P)	283	1.06
CUG(L)	194	0.42	CCG(P)	163	0.61
AUU(I)	1068	1.47	ACU(T)	565	1.71
AUC(I)	434	0.6	ACC(T)	223	0.68
AUA(I)	680	0.93	ACA(T)	392	1.19
AUG(M)	607	1	ACG(T)	140	0.42
GUU(V)	546	1.53	GCU(A)	622	1.82
GUC(V)	170	0.48	GCC(A)	202	0.59
GUA(V)	522	1.46	GCA(A)	412	1.2
GUG(V)	190	0.53	GCG(A)	133	0.39
UAU(Y)	762	1.63	UGU(C)	214	1.46
UAC(Y)	173	0.37	UGC(C)	80	0.54
UAA(*)	46	1.68	UGA(*)	17	0.62
UAG(*)	19	0.7	UGG(W)	464	1
CAU(H)	476	1.49	CGU(R)	338	1.33
CAC(H)	163	0.51	CGC(R)	106	0.42
CAA(Q)	711	1.55	CGA(R)	369	1.45

Codon	Number	RSCU	Codon	Number	RSCU
CAG(Q)	204	0.45	CGG(R)	109	0.43
AAU(N)	950	1.53	AGU(S)	422	1.26
AAC(N)	288	0.47	AGC(S)	114	0.34
AAA(K)	1048	1.52	AGA(R)	457	1.8
AAG(K)	333	0.48	AGG(R)	148	0.58
GAU(D)	844	1.6	GGU(G)	567	1.29
GAC(D)	212	0.4	GGC(G)	169	0.39
GAA(E)	1002	1.49	GGA(G)	705	1.61
GAG(E)	340	0.51	GGG(G)	314	0.72

g Relative synonymous codon usage (RSCU) in the chloroplast genome of *Sedum makinoi*.

Codon	Number	RSCU	Codon	Number	RSCU
UUU(F)	996	1.36	UCU(S)	540	1.65
UUC(F)	473	0.64	UCC(S)	298	0.91
UUA(L)	894	1.94	UCA(S)	406	1.24
UUG(L)	523	1.13	UCG(S)	196	0.6
CUU(L)	592	1.28	CCU(P)	401	1.51
CUC(L)	165	0.36	CCC(P)	220	0.83
CUA(L)	404	0.88	CCA(P)	280	1.05
CUG(L)	189	0.41	CCG(P)	161	0.61
AUU(I)	1063	1.47	ACU(T)	553	1.69
AUC(I)	427	0.59	ACC(T)	215	0.66
AUA(I)	685	0.94	ACA(T)	402	1.23
AUG(M)	598	1	ACG(T)	141	0.43
GUU(V)	533	1.51	GCU(A)	597	1.77
GUC(V)	179	0.51	GCC(A)	200	0.59
GUA(V)	515	1.46	GCA(A)	413	1.22
GUG(V)	186	0.53	GCG(A)	142	0.42
UAU(Y)	761	1.63	UGU(C)	214	1.47
UAC(Y)	170	0.37	UGC(C)	78	0.53
UAA(*)	50	1.74	UGA(*)	17	0.59
UAG(*)	19	0.66	UGG(W)	443	1
CAU(H)	469	1.53	CGU(R)	332	1.34
CAC(H)	145	0.47	CGC(R)	115	0.46
CAA(Q)	701	1.54	CGA(R)	356	1.43

Codon	Number	RSCU	Codon	Number	RSCU
CAG(Q)	207	0.46	CGG(R)	106	0.43
AAU(N)	932	1.52	AGU(S)	413	1.26
AAC(N)	291	0.48	AGC(S)	111	0.34
AAA(K)	1044	1.53	AGA(R)	432	1.74
AAG(K)	319	0.47	AGG(R)	149	0.6
GAU(D)	827	1.6	GGU(G)	569	1.32
GAC(D)	205	0.4	GGC(G)	158	0.37
GAA(E)	992	1.49	GGA(G)	691	1.6
GAG(E)	339	0.51	GGG(G)	307	0.71

h Relative synonymous codon usage (RSCU) in the chloroplast genome of *Sedum palmeri*.

Codon	Number	RSCU	Codon	Number	RSCU
UUU(F)	912	1.15	UCU(S)	419	1.47
UUC(F)	679	0.85	UCC(S)	292	1.02
UUA(L)	750	1.35	UCA(S)	396	1.39
UUG(L)	701	1.27	UCG(S)	236	0.83
CUU(L)	593	1.07	CCU(P)	262	1.1
CUC(L)	341	0.62	CCC(P)	202	0.85
CUA(L)	524	0.95	CCA(P)	285	1.19
CUG(L)	415	0.75	CCG(P)	206	0.86
AUU(I)	930	1.17	ACU(T)	310	1.2
AUC(I)	635	0.8	ACC(T)	199	0.77
AUA(I)	824	1.03	ACA(T)	351	1.36
AUG(M)	718	1	ACG(T)	175	0.68
GUU(V)	492	1.28	GCU(A)	271	1.42
GUC(V)	249	0.65	GCC(A)	137	0.72
GUA(V)	476	1.24	GCA(A)	231	1.21
GUG(V)	319	0.83	GCG(A)	127	0.66
UAU(Y)	632	1.28	UGU(C)	248	1.19
UAC(Y)	359	0.72	UGC(C)	168	0.81
UAA(*)	455	1.01	UGA(*)	403	0.9
UAG(*)	491	1.09	UGG(W)	451	1
CAU(H)	311	1.21	CGU(R)	139	0.65
CAC(H)	204	0.79	CGC(R)	91	0.43
CAA(Q)	574	1.25	CGA(R)	247	1.16

Codon	Number	RSCU	Codon	Number	RSCU
CAG(Q)	344	0.75	CGG(R)	192	0.9
AAU(N)	691	1.3	AGU(S)	245	0.86
AAC(N)	374	0.7	AGC(S)	124	0.43
AAA(K)	1001	1.27	AGA(R)	377	1.77
AAG(K)	577	0.73	AGG(R)	229	1.08
GAU(D)	491	1.34	GGU(G)	346	1.07
GAC(D)	244	0.66	GGC(G)	161	0.5
GAA(E)	718	1.34	GGA(G)	457	1.41
GAG(E)	353	0.66	GGG(G)	333	1.03

i Identified significantly variable codons (SVCs) among the eight *Sedum* plastomes.

Codon	Taxa
CTT(L)	*Sedum burrito*
ATA(I)	*Sedum burrito*, *Sedum palmeri*
TCC(S)	*Sedum burrito*, *Sedum palmeri*
TCG(S)	*Sedum burrito*
AGT(S)	*Sedum burrito*, *Sedum palmeri*
CCT(P)	*Sedum burrito*
CCG(P)	*Sedum burrito*
ACT(T)	*Sedum burrito*
GCT(A)	*Sedum burrito*
GCG(A)	*Sedum burrito*
CGT(R)	*Sedum burrito*, *Sedum palmeri*
CGG(R)	*Sedum burrito*
AGG(R)	*Sedum burrito*, *Sedum palmeri*
GGT(G)	*Sedum burrito*
GGG(G)	*Sedum burrito*, *Sedum palmeri*
TAG(*)	*Sedum burrito*, *Sedum palmeri*

Table S5

SSRs analysis of the eight *Sedum* plastomes.

SSR type	Repeat unit	Amount (Ratio%)							
		S. alfredii	***S. burrito***	***S. clavatum***	***S. dasyphyllum***	***S. furfuraceum***	***S. hernandezii***	***S. palmeri***	***S. makinoi***
**mono-**	A/T	102 (99.03)	103 (96.26)	101 (99.02)	102 (96.23)	85 (96.59)	104 (97.20)	91 (97.85)	103 (99.04)
	C/G	1 (0.97)	4 (3.74)	1 (0.98)	4 (3.77)	3 (3.41)	3 (2.80)	2 (2.15)	1 (0.96)
**di-**	AG/CT	6 (24.00)	10 (30.30)	8 (28.57)	9 (26.47)	10 (31.25)	10 (31.25)	10 (33.33)	6 (24.00)
	AT/AT	18 (72.00)	23 (69.70)	20 (71.43)	25 (73.53)	22 (68.75)	22 (68.75)	20 (66.67)	19 (76.00)
	CG/CG	1 (4.00)	0	0	0	0	0	0	0
**tri-**	AAG/CTT	1 (50.00)	1 (50.00)	1 (100.00)	0	1 (100.00)	1 (50.00)	1 (50.00)	1 (16.67)
	AAT/ATT	1 (50.00)	1 (50.00)	0	2 (100.00)	0	1 (50.00)	1 (50.00)	5 (83.33)
**tetra-**	AAAG/CTTT	0	1 (16.67)	1 (16.67)	1 (16.67)	1 (16.67)	1 (14.29)	1 (14.29)	0
	AAAT/ATTT	4 (57.14)	2 (33.33)	2 (33.33)	2 (33.33)	3 (50.00)	4 (57.14)	4 (57.14)	5 (83.33)
	AATC/ATTG	0	0	0	0	0	0	0	0
	AATG/ATTC	1 (14.29)	1 (16.67)	1 (16.67)	0	0	1 (14.29)	1 (14.29)	0
	ACAG/CTGT	1 (14.29)	1 (16.67)	1 (16.67)	1 (16.67)	1 (16.67)	1 (14.29)	1 (14.29)	1 (16.67)
	AATT/AATT	0	1 (16.67)	1 (16.67)	0	1 (16.67)	0	0	0
	AGAT/ATCT	0	0	0	2 (33.33)	0	0	0	0
	AACG/CGTT	1 (14.29)	0	0	0	0	0	0	0
**penta-**	AAAAT/ATTTT	0	1 (100.00)	0	0	2 (100.00)	1 (100.00)	0	0
	AAGAT/ATCTT	0	0	0	0	0	0	1 (100.00)	0
	AATAT/ATATT	0	0	0	0	0	0	0	0
**hexa-**	ACATAT/ATATGT	0	1 (100.00)	0	0	0	0	0	0
	**total**	137	150	137	148	129	149	133	141

a Detailed analysis of simple sequence repeats (SSRs) identified within the chloroplast genome of *Sedum alfredii*.

**Repeats**	**3**	**4**	**5**	**6**	**7**	**8**	**9**	**10**	**11**	**12**	**13**	**14**	**15**	**16**	**17**	**18**	**total**
A/T	-	-	-	-	-	39	26	19	14	7	1	-	-	-	-	1	107
C/G	-	-	-	-	-	-	-	-	-	-	-	-	-	-	-	-	1
AG/CT	-	10	-	-	-	-	-	-	-	-	-	-	-	-	-	-	10
AT/AT	-	16	2	1	1	-	-	-	-	-	-	-	-	-	-	-	20
CG/CG	-	1	-	-	-	-	-	-	-	-	-	-	-	-	-	-	1
AAG/CTT	-	2	-	-	-	-	-	-	-	-	-	-	-	-	-	-	2
AAT/ATT	-	1	-	-	-	-	-	-	-	-	-	-	-	-	-	-	1
AAAT/ATTT	3	-	1	-	-	-	-	-	-	-	-	-	-	-	-	-	4
AACG/CGTT	1	-	-	-	-	-	-	-	-	-	-	-	-	-	-	-	1
AATG/ATTC	1	-	-	-	-	-	-	-	-	-	-	-	-	-	-	-	1
ACAG/CTGT	1	-	-	-	-	-	-	-	-	-	-	-	-	-	-	-	1

b Detailed analysis of simple sequence repeats (SSRs) identified within the chloroplast genome of *Sedum burrito*.

**Repeats**	**3**	**4**	**5**	**6**	**7**	**8**	**9**	**10**	**11**	**12**	**13**	**14**	**15**	**16**	**17**	**total**
A/T	-	-	-	-	-	47	32	13	4	5	3	3	-	1	1	109
C/G	-	-	-	-	-	5	1	-	-	-	-	-	-	-	-	6
AG/CT	-	16	-	-	-	-	-	-	-	-	-	-	-	-	-	16
AT/AT	-	14	8	2	1	-	-	-	-	-	-	-	-	-	-	25
AAG/CTT	-	2	-	-	-	-	-	-	-	-	-	-	-	-	-	2
AAT/ATT	-	1	-	-	-	-	-	-	-	-	-	-	-	-	-	1
AAAG/CTTT	1	-	-	-	-	-	-	-	-	-	-	-	-	-	-	1
AAAT/ATTT	2	-	-	-	-	-	-	-	-	-	-	-	-	-	-	2
AATG/ATTC	1	-	-	-	-	-	-	-	-	-	-	-	-	-	-	1
AATT/AATT	1	-	-	-	-	-	-	-	-	-	-	-	-	-	-	1
ACAG/CTGT	1	-	-	-	-	-	-	-	-	-	-	-	-	-	-	1
AAAAT/ATTTT	1	-	-	-	-	-	-	-	-	-	-	-	-	-	-	1
ACATAT/ATATGT	1	-	-	-	-	-	-	-	-	-	-	-	-	-	-	1

c Detailed analysis of simple sequence repeats (SSRs) identified within the chloroplast genome of *Sedum clavatum*.

**Repeats**	**3**	**4**	**5**	**6**	**7**	**8**	**9**	**10**	**11**	**12**	**13**	**14**	**15**	**total**
A/T	-	-	-	-	-	40	30	19	7	6	4	1	1	108
C/G	-	-	-	-	-	2	-	-	-	-	-	-	-	2
AG/CT	-	13	-	-	-	-	-	-	-	-	-	-	-	13
AT/AT	-	16	4	1	1	-	-	-	-	-	-	-	-	22
AAG/CTT	-	2	-	-	-	-	-	-	-	-	-	-	-	2
AAAG/CTTT	1	-	-	-	-	-	-	-	-	-	-	-	-	1
AAAT/ATTT	2	-	-	-	-	-	-	-	-	-	-	-	-	2
AATG/ATTC	1	-	-	-	-	-	-	-	-	-	-	-	-	1
AATT/AATT	1	-	-	-	-	-	-	-	-	-	-	-	-	1
ACAG/CTGT	1	-	-	-	-	-	-	-	-	-	-	-	-	1

d Detailed analysis of simple sequence repeats (SSRs) identified within the chloroplast genome of *Sedum dasyphyllum*.

**Repeats**	**3**	**4**	**5**	**6**	**7**	**8**	**9**	**10**	**11**	**12**	**13**	**14**	**15**	**16**	**17**	**18**	**19**	**total**
A/T	-	-	-	-	-	40	41	11	6	3	4	-	1	-	-	-	1	107
C/G	-	-	-	-	-	5	-	-	1	-	-	-	-	-	-	-	-	6
AG/CT	-	15	-	-	-	-	-	-	-	-	-	-	-	-	-	-	-	15
AT/AT	-	17	8	2	-	-	-	-	-	-	-	-	-	-	-	-	-	27
AAT/ATT	-	2	-	-	-	-	-	-	-	-	-	-	-	-	-	-	-	2
AAAG/CTTT	1	-	-	-	-	-	-	-	-	-	-	-	-	-	-	-	-	1
AAAT/ATTT	2	-	-	-	-	-	-	-	-	-	-	-	-	-	-	-	-	2
ACAG/CTGT	1	-	-	-	-	-	-	-	-	-	-	-	-	-	-	-	-	1
AGAT/ATCT	2	-	-	-	-	-	-	-	-	-	-	-	-	-	-	-	-	2

e Detailed analysis of simple sequence repeats (SSRs) identified within the chloroplast genome of *Sedum furfuraceum*.

**Repeats**	**3**	**4**	**5**	**6**	**7**	**8**	**9**	**10**	**11**	**12**	**13**	**14**	**total**
A/T	-	-	-	-	-	32	29	7	11	6	3	2	90
C/G	-	-	-	-	-	5	-	-	-	-	-	-	5
AG/CT	-	16	-	-	-	-	-	-	-	-	-	-	16
AT/AT	-	17	4	2	1	-	-	-	-	-	-	-	24
AAG/CTT	-	2	-	-	-	-	-	-	-	-	-	-	2
AAAG/CTTT	1	-	-	-	-	-	-	-	-	-	-	-	1
AAAT/ATTT	3	-	-	-	-	-	-	-	-	-	-	-	3
AATT/AATT	1	-	-	-	-	-	-	-	-	-	-	-	1
ACAG/CTGT	1	-	-	-	-	-	-	-	-	-	-	-	1
AAAAT/ATTTT	2	-	-	-	-	-	-	-	-	-	-	-	2

f Detailed analysis of simple sequence repeats (SSRs) identified within the chloroplast genome of *Sedum hernandezii*.

**Repeats**	**3**	**4**	**5**	**6**	**7**	**8**	**9**	**10**	**11**	**12**	**13**	**14**	**15**	**16**	**17**	**18**	**19**	**20**	**21**	**22**	**total**
A/T	-	-	-	-	-	40	32	18	9	5	1	1	-	-	2	1	-	-	-	1	110
C/G	-	-	-	-	-	4	1	-	-	-	-	-	-	-	-	-	-	-	-	-	5
AG/CT	-	16	-	-	-	-	-	-	-	-	-	-	-	-	-	-	-	-	-	-	16
AT/AT	-	19	4	-	1	-	-	-	-	-	-	-	-	-	-	-	-	-	-	-	24
AAG/CTT	-	2	-	-	-	-	-	-	-	-	-	-	-	-	-	-	-	-	-	-	2
AAT/ATT	-	1	-	-	-	-	-	-	-	-	-	-	-	-	-	-	-	-	-	-	1
AAAG/CTTT	1	-	-	-	-	-	-	-	-	-	-	-	-	-	-	-	-	-	-	-	1
AAAT/ATTT	4	-	-	-	-	-	-	-	-	-	-	-	-	-	-	-	-	-	-	-	4
AATG/ATTC	-	1	-	-	-	-	-	-	-	-	-	-	-	-	-	-	-	-	-	-	1
ACAG/CTGT	1	-	-	-	-	-	-	-	-	-	-	-	-	-	-	-	-	-	-	-	1
AAAAT/ATTTT	1	-	-	-	-	-	-	-	-	-	-	-	-	-	-	-	-	-	-	-	1

g Detailed analysis of simple sequence repeats (SSRs) identified within the chloroplast genome of *Sedum makinoi*.

**Repeats**	**3**	**4**	**5**	**6**	**7**	**8**	**9**	**10**	**11**	**12**	**13**	**14**	**total**
A/T	-	-	-	-	-	46	30	18	8	4	1	2	109
C/G	-	-	-	-	-	1	-	-	-	-	-	-	1
AG/CT	-	10	-	-	-	-	-	-	-	-	-	-	10
AT/AT	-	15	2	2	1	-	1	-	-	-	-	-	21
AAG/CTT	-	2	-	-	-	-	-	-	-	-	-	-	2
AAT/ATT	-	5	-	-	-	-	-	-	-	-	-	-	5
AAAT/ATTT	5	-	-	-	-	-	-	-	-	-	-	-	5
ACAG/CTGT	1	-	-	-	-	-	-	-	-	-	-	-	1

h Detailed analysis of simple sequence repeats (SSRs) identified within the chloroplast genome of *Sedum palmeri*.

Repeats	3	4	5	6	7	8	9	10	11	12	13	14	total
A/T	-	-	-	-	-	41	31	18	4	1	1	1	97
C/G	-	-	-	-	-	4	-	-	-	-	-	-	4
AG/CT	-	16	-	-	-	-	-	-	-	-	-	-	16
AT/AT	-	16	4	1	-	-	1	-	-	-	-	-	22
AAG/CTT	-	2	-	-	-	-	-	-	-	-	-	-	2
AAT/ATT	-	1	-	-	-	-	-	-	-	-	-	-	1
AAAG/CTTT	1	-	-	-	-	-	-	-	-	-	-	-	1
AAAT/ATTT	4	-	-	-	-	-	-	-	-	-	-	-	4
AATG/ATTC	1	-	-	-	-	-	-	-	-	-	-	-	1
ACAG/CTGT	1	-	-	-	-	-	-	-	-	-	-	-	1
AAGAT/ATCTT	1	-	-	-	-	-	-	-	-	-	-	-	1

Table S6

Predictive RNA editing sites in the eight *Sedum* plastomes.

a Putative RNA editing sites in the *Sedum alfredii* chloroplast genome.

Gene	Nucleotide positions	Amino Acid positions	Codon and Amino Acid conversion	Score
accD	989	330	GCA(A)=>GTA(V)	1
	1376	459	CCT(P)=>CTT(L)	1
atpB	1487	496	TCG(S)=>TTG(L)	1
atpF	92	31	CCA(P)=>CTA(L)	0.86
clpP	556	186	CAC(H)=>TAC(Y)	1
matK	637	213	CAT(H)=>TAT(Y)	1
ndhA	341	114	TCA(S)=>TTA(L)	1
	566	189	TCA(S)=>TTA(L)	1
ndhB	149	50	TCA(S)=>TTA(L)	1
	586	196	CAT(H)=>TAT(Y)	1
	611	204	TCA(S)=>TTA(L)	0.8
	737	246	CCA(P)=>CTA(L)	1
	746	249	TCT(S)=>TTT(F)	1
	830	277	TCA(S)=>TTA(L)	1
	836	279	TCA(S)=>TTA(L)	1
	1255	419	CAT(H)=>TAT(Y)	1
	1481	494	CCA(P)=>CTA(L)	1
ndhD	35	12	GCT(A)=>GTT(V)	1
	154	52	CGT(R)=>TGT(C)	1
ndhF	290	97	TCA(S)=>TTA(L)	1
	586	196	CTT(L)=>TTT(F)	0.8

Gene	Nucleotide positions	Amino Acid positions	Codon and Amino Acid conversion	Score
	2201	734	TCT(S)=>TTT(F)	1
ndhG	166	56	CAT(H)=>TAT(Y)	0.8
	314	105	ACA(T)=>ATA(I)	0.8
petB	617	206	CCA(P)=>CTA(L)	1
rpoA	833	278	TCA(S)=>TTA(L)	1
rpoB	338	113	TCT(S)=>TTT(F)	1
	551	184	TCA(S)=>TTA(L)	1
	566	189	CCA(P)=>CTA(L)	1
	2426	809	TCA(S)=>TTA(L)	0.86
rpoC1	41	14	TCA(S)=>TTA(L)	1
	1492	498	CGT(R)=>TGT(C)	1
	1991	664	ACC(T)=>ATC(I)	0.86
rpoC2	1319	440	GCT(A)=>GTT(V)	1
	2263	755	CCG(P)=>TCG(S)	1
	3262	1088	CCG(P)=>TCG(S)	1
	3731	1244	TCA(S)=>TTA(L)	0.86
	4100	1367	TCC(S)=>TTC(F)	0.8
rps14	80	27	TCA(S)=>TTA(L)	1
	149	50	CCG(P)=>CTG(L)	1
rps16	212	71	TCA(S)=>TTA(L)	0.83

b Putative RNA editing sites in the *Sedum burrito* chloroplast genome.

Gene	Nucleotide positions	Amino Acid positions	Codon and Amino Acid conversion	Score
accD	983	328	GCA (A) => GTA (V)	1
	1370	457	CCT (P) => CTT (L)	1
atpB	1487	496	TCG (S) => TTG (L)	1
atpF	92	31	CCA (P) => CTA (L)	0.86
clpP	556	186	CAC (H) => TAC (Y)	1
matK	637	213	CAT (H) => TAT (Y)	1
	1178	393	TCA (S) => TTA (L)	0.86
ndhA	341	114	TCA (S) => TTA (L)	1
	566	189	TCA (S) => TTA (L)	1
ndhB	149	50	TCA (S) => TTA (L)	1
	542	181	ACG (T) => ATG (M)	1
	586	196	CAT (H) => TAT (Y)	1
	611	204	TCA (S) => TTA (L)	0.8
	737	246	CCA (P) => CTA (L)	1
	746	249	TCT (S) => TTT (F)	1
	830	277	TCA (S) => TTA (L)	1
	836	279	TCA (S) => TTA (L)	1
	1255	419	CAT (H) => TAT (Y)	1
	1481	494	CCA (P) => CTA (L)	1
ndhD	32	11	TCA (S) => TTA (L)	1
ndhF	290	97	TCA (S) => TTA (L)	1
	586	196	CTT (L) => TTT (F)	0.8
	2201	734	TCT (S) => TTT (F)	1
ndhG	166	56	CAT (H) => TAT (Y)	0.8

Gene	Nucleotide positions	Amino Acid positions	Codon and Amino Acid conversion	Score
	314	105	ACA (T) => ATA (I)	0.8
petB	617	206	CCA (P) => CTA (L)	1
psbF	77	26	TCT (S) => TTT (F)	1
rpoA	824	275	TCA (S) => TTA (L)	1
rpoB	338	113	TCT (S) => TTT (F)	1
	551	184	TCA (S) => TTA (L)	1
	566	189	CCA (P) => CTA (L)	1
	2426	809	TCA (S) => TTA (L)	0.86
rpoC1	41	14	TCA (S) => TTA (L)	1
	1492	498	CGT (R) => TGT (C)	1
	1991	664	ACC (T) => ATC (I)	0.86
rpoC2	2263	755	CCG (P) => TCG (S)	1
	3233	1078	GCA (A) => GTA (V)	0.86
	3734	1245	TCA (S) => TTA (L)	0.86
	4109	1370	TCC (S) => TTC (F)	0.8
rps14	80	27	TCA (S) => TTA (L)	1
rps16	212	71	TCA (S) => TTA (L)	0.83

c Putative RNA editing sites in the *Sedum clavatum* chloroplast genome.

Gene	Nucleotide positions	Amino Acid positions	Codon and Amino Acid conversion	Score
accD	983	328	GCA(A)=>GTA(V)	1
	1370	457	CCT(P)=>CTT(L)	1
atpB	1487	496	TCG(S)=>TTG(L)	1
atpF	92	31	CCA(P)=>CTA(L)	0.86
clpP	556	186	CAC(H)=>TAC(Y)	1
matK	637	213	CAT(H)=>TAT(Y)	1
	1178	393	TCA(S)=>TTA(L)	0.86
ndhA	341	114	TCA(S)=>TTA(L)	1
	566	189	TCA(S)=>TTA(L)	1
ndhB	149	50	TCA(S)=>TTA(L)	1
	542	181	ACG(T)=>ATG(M)	1
	586	196	CAT(H)=>TAT(Y)	1
	611	204	TCA(S)=>TTA(L)	0.8
	737	246	CCA(P)=>CTA(L)	1
	746	249	TCT(S)=>TTT(F)	1
	830	277	TCA(S)=>TTA(L)	1
	836	279	TCA(S)=>TTA(L)	1
	1255	419	CAT(H)=>TAT(Y)	1
	1481	494	CCA(P)=>CTA(L)	1
ndhD	47	16	TCT(S)=>TTT(F)	0.8
	154	52	CGT(R)=>TGT(C)	1
	383	128	TCA(S)=>TTA(L)	1
ndhF	290	97	TCA(S)=>TTA(L)	1
	586	196	CTT(L)=>TTT(F)	0.8

Gene	Nucleotide positions	Amino Acid positions	Codon and Amino Acid conversion	Score
	2207	736	TCT(S)=>TTT(F)	1
ndhG	166	56	CAT(H)=>TAT(Y)	0.8
	314	105	ACA(T)=>ATA(I)	0.8
petB	611	204	CCA(P)=>CTA(L)	1
psbF	77	26	TCT(S)=>TTT(F)	1
rpoA	824	275	TCA(S)=>TTA(L)	1
rpoB	338	113	TCT(S)=>TTT(F)	1
	551	184	TCA(S)=>TTA(L)	1
	566	189	CCA(P)=>CTA(L)	1
	2426	809	TCA(S)=>TTA(L)	0.86
rpoC1	41	14	TCA(S)=>TTA(L)	1
rpoC2	2263	755	CCG(P)=>TCG(S)	1
	3224	1075	GCA(A)=>GTA(V)	0.86
	3725	1242	TCA(S)=>TTA(L)	0.86
	4100	1367	TCC(S)=>TTC(F)	0.8
rps14	80	27	TCA(S)=>TTA(L)	1
rps16	212	71	TCA(S)=>TTA(L)	0.83

d Putative RNA editing sites in the *Sedum dasyphyllum* chloroplast genome.

Gene	Nucleotide positions	Amino Acid positions	Codon and Amino Acid conversion	Score
accD	13	5	CGG(R)=>TGG(W)	1
	977	326	GCA(A)=>GTA(V)	1
	1364	455	CCT(P)=>CTT(L)	1
	1405	469	CTC(L)=>TTC(F)	1
atpB	1487	496	TCG(S)=>TTG(L)	1
atpF	92	31	CCA(P)=>CTA(L)	0.86
atpI	620	207	TCA(S)=>TTA(L)	1
clpP	556	186	CAC(H)=>TAC(Y)	1
matK	631	211	CAT(H)=>TAT(Y)	1
ndhA	341	114	TCA(S)=>TTA(L)	1
	566	189	TCA(S)=>TTA(L)	1
ndhB	64	22	CTT(L)=>TTT(F)	1
	149	50	TCA(S)=>TTA(L)	1
	542	181	ACG(T)=>ATG(M)	1
	586	196	CAT(H)=>TAT(Y)	1
	611	204	TCA(S)=>TTA(L)	0.8
	737	246	CCA(P)=>CTA(L)	1
	746	249	TCT(S)=>TTT(F)	1
	830	277	TCA(S)=>TTA(L)	1
	836	279	TCA(S)=>TTA(L)	1
	1255	419	CAT(H)=>TAT(Y)	1
	1481	494	CCA(P)=>CTA(L)	1
ndhD	35	12	GCT(A)=>GTT(V)	1
	47	16	TCT(S)=>TTT(F)	0.8

Gene	Nucleotide positions	Amino Acid positions	Codon and Amino Acid conversion	Score
	154	52	CGT(R)=>TGT(C)	1
	383	128	TCG(S)=>TTG(L)	1
	1310	437	TCA(S)=>TTA(L)	0.8
ndhF	290	97	TCA(S)=>TTA(L)	1
	586	196	CTT(L)=>TTT(F)	0.8
	2201	734	TCT(S)=>TTT(F)	1
ndhG	131	44	GCT(A)=>GTT(V)	1
	166	56	CAT(H)=>TAT(Y)	0.8
	314	105	ACA(T)=>ATA(I)	0.8
petB	611	204	CCA(P)=>CTA(L)	1
petG	14	5	TCT(S)=>TTT(F)	0.86
psaI	28	10	CTT(L)=>TTT(F)	0.86
psbF	77	26	TCT(S)=>TTT(F)	1
rpoA	827	276	TCA(S)=>TTA(L)	1
rpoB	338	113	TCT(S)=>TTT(F)	1
	551	184	TCA(S)=>TTA(L)	1
	566	189	TCA(S)=>TTA(L)	1
	2426	809	TCA(S)=>TTA(L)	0.86
rpoC1	41	14	TCA(S)=>TTA(L)	1
	1447	483	CGT(R)=>TGT(C)	1
	1946	649	ACC(T)=>ATC(I)	0.86
rpoC2	1552	518	CCG(P)=>TCG(S)	0.86
	3230	1077	GCA(A)=>GTA(V)	0.86
	3731	1244	TCA(S)=>TTA(L)	0.86
	4106	1369	TCC(S)=>TTC(F)	0.8

Gene	Nucleotide positions	Amino Acid positions	Codon and Amino Acid conversion	Score
rps14	80	27	TCA(S)=>TTA(L)	1
	149	50	CCG(P)=>CTG(L)	1
rps16	212	71	TCA(S)=>TTA(L)	0.83

e Putative RNA editing sites in the *Sedum furfuraceum* chloroplast genome.

Gene	Nucleotide positions	Amino Acid positions	Codon and Amino Acid conversion	Score
accD	983	328	GCA(A)=>GTA(V)	1
	1370	457	CCT(P)=>CTT(L)	1
atpB	1487	496	TCG(S)=>TTG(L)	1
clpP	556	186	CAC(H)=>TAC(Y)	1
matK	637	213	CAT(H)=>TAT(Y)	1
	1178	393	TCA(S)=>TTA(L)	0.86
ndhA	341	114	TCA(S)=>TTA(L)	1
	566	189	TCA(S)=>TTA(L)	1
ndhB	149	50	TCA(S)=>TTA(L)	1
	542	181	ACG(T)=>ATG(M)	1
	586	196	CAT(H)=>TAT(Y)	1
	611	204	TCA(S)=>TTA(L)	0.8
	737	246	CCA(P)=>CTA(L)	1
	746	249	TCT(S)=>TTT(F)	1
	830	277	TCA(S)=>TTA(L)	1
	836	279	TCA(S)=>TTA(L)	1
	1255	419	CAT(H)=>TAT(Y)	1
	1481	494	CCA(P)=>CTA(L)	1
ndhD	47	16	TCT(S)=>TTT(F)	0.8
	154	52	CGT(R)=>TGT(C)	1
	383	128	TCA(S)=>TTA(L)	1
ndhF	290	97	TCA(S)=>TTA(L)	1
	586	196	CTT(L)=>TTT(F)	0.8
	2216	739	TCT(S)=>TTT(F)	1

Gene	Nucleotide positions	Amino Acid positions	Codon and Amino Acid conversion	Score
ndhG	166	56	CAT(H)=>TAT(Y)	0.8
	314	105	ACA(T)=>ATA(I)	0.8
petB	611	204	CCA(P)=>CTA(L)	1
psbF	77	26	TCT(S)=>TTT(F)	1
rpoA	824	275	TCA(S)=>TTA(L)	1
rpoB	338	113	TCT(S)=>TTT(F)	1
	551	184	TCA(S)=>TTA(L)	1
	566	189	CCA(P)=>CTA(L)	1
rpoC1	41	14	TCA(S)=>TTA(L)	1
rpoC2	2084	695	GCA(A)=>GTA(V)	1
	2263	755	CCG(P)=>TCG(S)	1
	2644	882	CCC(P)=>TCC(S)	0.86
	3233	1078	GCA(A)=>GTA(V)	0.86
	3734	1245	TCA(S)=>TTA(L)	0.86
	4109	1370	TCC(S)=>TTC(F)	0.8
rps14	80	27	TCA(S)=>TTA(L)	1
rps16	212	71	TCA(S)=>TTA(L)	0.83

f Putative RNA editing sites in the *Sedum hernandezii* chloroplast genome.

Gene	Nucleotide positions	Amino Acid positions	Codon and Amino Acid conversion	Score
accD	983	328	GCA(A)=>GTA(V)	1
	1370	457	CCT(P)=>CTT(L)	1
atpB	1487	496	TCG(S)=>TTG(L)	1
atpF	92	31	CCA(P)=>CTA(L)	0.86
clpP	556	186	CAC(H)=>TAC(Y)	1
matK	637	213	CAT(H)=>TAT(Y)	1
	1178	393	TCA(S)=>TTA(L)	0.86
ndhA	341	114	TCA(S)=>TTA(L)	1
	566	189	TCA(S)=>TTA(L)	1
ndhB	149	50	TCA(S)=>TTA(L)	1
	542	181	ACG(T)=>ATG(M)	1
	586	196	CAT(H)=>TAT(Y)	1
	611	204	TCA(S)=>TTA(L)	0.8
	737	246	CCA(P)=>CTA(L)	1
	746	249	TCT(S)=>TTT(F)	1
	830	277	TCA(S)=>TTA(L)	1
	836	279	TCA(S)=>TTA(L)	1
	1255	419	CAT(H)=>TAT(Y)	1
	1481	494	CCA(P)=>CTA(L)	1
ndhD	47	16	TCT(S)=>TTT(F)	0.8
	154	52	CGT(R)=>TGT(C)	1
	383	128	TCA(S)=>TTA(L)	1
	1148	383	ACG(T)=>ATG(M)	1
ndhF	290	97	TCA(S)=>TTA(L)	1

	586	196	CTT(L)=>TTT(F)	0.8
	2201	734	TCT(S)=>TTT(F)	1
ndhG	166	56	CAT(H)=>TAT(Y)	0.8
	314	105	ACA(T)=>ATA(I)	0.8
petB	611	204	CCA(P)=>CTA(L)	1
psbF	77	26	TCT(S)=>TTT(F)	1
rpoB	338	113	TCT(S)=>TTT(F)	1
	551	184	TCA(S)=>TTA(L)	1
	566	189	CCA(P)=>CTA(L)	1
	2426	809	TCA(S)=>TTA(L)	0.86
rpoC1	41	14	TCA(S)=>TTA(L)	1
	1447	483	CGT(R)=>TGT(C)	1
	1946	649	ACC(T)=>ATC(I)	0.86
rpoC2	2251	751	CCG(P)=>TCG(S)	1
	3185	1062	ACA(T)=>ATA(I)	0.86
	3215	1072	GCA(A)=>GTA(V)	0.86
	3716	1239	TCA(S)=>TTA(L)	0.86
rps14	80	27	TCA(S)=>TTA(L)	1
rps16	212	71	TCA(S)=>TTA(L)	0.83

g Putative RNA editing sites in the *Sedum makinoi* chloroplast genome.

Gene	Nucleotide positions	Amino Acid positions	Codon and Amino Acid conversion	Score
accD	938	313	GCA(A)=>GTA(V)	1
	1325	442	CCT(P)=>CTT(L)	1
atpF	98	33	CCA(P)=>CTA(L)	0.86
atpI	623	208	GCG(A)=>GTG(V)	0.86
clpP	556	186	CAC(H)=>TAC(Y)	1
matK	631	211	CAT(H)=>TAT(Y)	1
ndhA	341	114	TCA(S)=>TTA(L)	1
	566	189	TCA(S)=>TTA(L)	1
ndhB	149	50	TCA(S)=>TTA(L)	1
	586	196	CAT(H)=>TAT(Y)	1
	611	204	TCA(S)=>TTA(L)	0.8
	737	246	CCA(P)=>CTA(L)	1
	746	249	TCT(S)=>TTT(F)	1
	830	277	TCA(S)=>TTA(L)	1
	836	279	TCA(S)=>TTA(L)	1
	1255	419	CAT(H)=>TAT(Y)	1
	1481	494	CCA(P)=>CTA(L)	1
ndhD	35	12	GCT(A)=>GTT(V)	1
	154	52	CGT(R)=>TGT(C)	1
	383	128	TCA(S)=>TTA(L)	1
ndhF	290	97	TCA(S)=>TTA(L)	1
	586	196	CTT(L)=>TTT(F)	0.8
ndhG	166	56	CAT(H)=>TAT(Y)	0.8
	314	105	ACA(T)=>ATA(I)	0.8

petB	611	204	CCA(P)=>CTA(L)	1
rpoA	824	275	TCA(S)=>TTA(L)	1
rpoB	338	113	TCT(S)=>TTT(F)	1
	551	184	TCA(S)=>TTA(L)	1
	566	189	CCA(P)=>CTA(L)	1
	2426	809	TCA(S)=>TTA(L)	0.86
rpoC1	41	14	TCA(S)=>TTA(L)	1
	1447	483	CGT(R)=>TGT(C)	1
	1946	649	ACC(T)=>ATC(I)	0.86
rpoC2	1319	440	GCT(A)=>GTT(V)	1
	2263	755	CCG(P)=>TCG(S)	1
	3262	1088	CCG(P)=>TCG(S)	1
	3731	1244	TCA(S)=>TTA(L)	0.86
	4106	1369	TCC(S)=>TTC(F)	0.8
rps14	80	27	TCA(S)=>TTA(L)	1
	149	50	CCG(P)=>CTG(L)	1
rps16	212	71	TCA(S)=>TTA(L)	0.83

h Putative RNA editing sites in the *Sedum palmeri* chloroplast genome.

Gene	Nucleotide positions	Amino Acid positions	Codon and Amino Acid conversion	Score
accD	983	328	GCA(A)=>GTA(V)	1
	1370	457	CCT(P)=>CTT(L)	1
atpB	1487	496	TCG(S)=>TTG(L)	1
atpF	92	31	CCA(P)=>CTA(L)	0.86
clpP	556	186	CAC(H)=>TAC(Y)	1
matK	637	213	CAT(H)=>TAT(Y)	1
	1178	393	TCA(S)=>TTA(L)	0.86
ndhA	341	114	TCA(S)=>TTA(L)	1
	566	189	TCA(S)=>TTA(L)	1
ndhB	149	50	TCA(S)=>TTA(L)	1
	542	181	ACG(T)=>ATG(M)	1
	586	196	CAT(H)=>TAT(Y)	1
	611	204	TCA(S)=>TTA(L)	0.8
	737	246	CCA(P)=>CTA(L)	1
	746	249	TCT(S)=>TTT(F)	1
	830	277	TCA(S)=>TTA(L)	1
	836	279	TCA(S)=>TTA(L)	1
	1255	419	CAT(H)=>TAT(Y)	1
	1481	494	CCA(P)=>CTA(L)	1
ndhD	47	16	TCT(S)=>TTT(F)	0.8
	154	52	CGT(R)=>TGT(C)	1
	383	128	TCA(S)=>TTA(L)	1
ndhF	290	97	TCA(S)=>TTA(L)	1
	586	196	CTT(L)=>TTT(F)	0.8

	2201	734	TCT(S)=>TTT(F)	1
ndhG	166	56	CAT(H)=>TAT(Y)	0.8
	314	105	ACA(T)=>ATA(I)	0.8
petB	178	60	CCA(P)=>TCA(S)	1
	188	63	GCC(A)=>GTC(V)	1
	1285	429	CCG(P)=>TCG(S)	1
psbF	77	26	TCT(S)=>TTT(F)	1
rpoA	824	275	TCA(S)=>TTA(L)	1
rpoB	338	113	TCT(S)=>TTT(F)	1
	551	184	TCA(S)=>TTA(L)	1
	566	189	CCA(P)=>CTA(L)	1
	2420	807	TCA(S)=>TTA(L)	0.86
rpoC1	41	14	TCA(S)=>TTA(L)	1
rpoC2	2263	755	CCG(P)=>TCG(S)	1
	2986	996	CCT(P)=>TCT(S)	0.86
	3233	1078	GCA(A)=>GTA(V)	0.86
	3734	1245	TCA(S)=>TTA(L)	0.86
	3986	1329	TCC(S)=>TTC(F)	0.86
	4109	1370	TCC(S)=>TTC(F)	0.8
rps14	80	27	TCA(S)=>TTA(L)	1
rps16	212	71	TCA(S)=>TTA(L)	0.83
